# Caecal microbiome transplant inhibits transmission and intestinal colonisation of *Campylobacter jejuni* in broiler chickens

**DOI:** 10.1186/s44364-026-00031-8

**Published:** 2026-06-15

**Authors:** Rachel Gilroy, Gemma Chaloner, Amy Wedley, Peter Richards-Rios, Sian Pottenger, Paul Wigley

**Affiliations:** 1 Institute of Infection Veterinary and Ecological Sciences, University of Liverpool, Liverpool, United Kingdom; 2 SRUC Veterinary Services, Scotland’s Rural College, Pentlands Science Park, Bush Loan, Penicuik, Midlothian UK; 3 Bristol Veterinary School, University of Bristol, Langford, Bristol, BS40 5DU UK

**Keywords:** Campylobacter, Microbiome, Microbiome transplant, Transmission, Microbial-based interventions

## Abstract

*Campylobacter jejuni* is the most frequent cause of foodborne bacterial gastroenteritis with poultry products the most frequent source of infection. *C. jejuni* can colonise the intestinal tract of the chicken and in particular the large blind caeca to a high level accompanied by faecal shedding and rapid transmission in flocks. As such, reducing transmission and intestinal colonisation in poultry meat production is considered a key target to reduce human infection. Whilst vaccines and feed-based approaches including modulation of the microbiome are considered most likely to reduce numbers in the chicken caeca, neither have yet shown the capacity to lead to significant reductions. We have previously shown that administration of a caecal microbiome transplant (CMT) at hatch acts to modify the microbiome, increasing diversity and reducing Enterobacteriacae levels associated with poor gut health and increased *Campylobacter* susceptibility. When challenged at 21 days old with *C. jejuni* M1 in a seeder bird infection model, birds in groups receiving CMT showed reduced transmission and significantly lower levels of *C. jejuni* at post-mortem examination at 35 days of age than control birds or birds treated with a commercial microflora competitive exclusion product (Aviguard). These data show that a microbiome-based intervention has the potential to inhibit *C. jejuni* transmission and decrease levels in the caeca at slaughter age. This is modelled to lead to a significant reduction in human cases. CMT offers a valuable tool to determine protective taxa in the chicken gut, aiding rational development of microbial interventions as well as a low-cost platform to help understand immunological development in the chicken gut.

## Introduction

*Campylobacter jejuni*, a highly motile Gram-negative proteobacteria, is the most frequent cause of human bacterial foodborne gastroenteritis worldwide with regarded as the major source of infection [16]. The European Food Safety Authority’s 2008 baseline survey showed *C. jejuni* on over 60% of retail chicken within the EU, but subsequent intervention strategies aimed at reducing *C.jejuni* burden within the commercial production have shown limited success towards reaching the levels of reduction considered to reduce human disease.(Hazards et al., 2020). Pragmatic and cost-effective means of large-scale on-farm control within poultry production without the use of antimicrobials is a public health priority. However, unlike *Salmonella*, where vaccination has proved successful as part of control strategies, the nature of both the pathogen and host response to *Campylobacter* in the chicken make the development of vaccines challenging [6, 21]. *C. jejuni* colonises the gastrointestinal tract of chickens to high levels, with colonisation of the two large blind caeca the main site of persistence, though colonisation of the small intestine and even the crop is found with certain strains [4, 17]. Whilst colonisation rarely presents as overt clinical disease, infection of chickens causes a marked intestinal inflammatory response that may be associated with poor gut health and decreased welfare in broiler chicken breeds [1, 2, 16]. We know that *C. jejuni* infection elicits both local mucosal adaptive and innate immune responses and it is thought that infection is largely limited to the gut via Th17 and antibody-mediated responses [30]. The intestinal microbiome is likely to act both as a barrier to *C. jejuni* colonisation and help drive the development of an effective mucosal immune system. As such, manipulation of the broiler chicken microbiome may offer a convenient and practical route to reduce *C. jejuni* within flocks.

Microbiome-based interventions in the chicken have a long history stretching back to the use of cultured avian intestinal flora to reduce *Salmonella* colonisation in chicks [29] which has formed the basis of many subsequent studies on probiotics. Any phenotypic changes following the manipulation of the chicken microbiome would broadly appear to be driven by two main possibilities. The first is a competitive exclusion (CE) effect, originally an ecological term, based around competition for a niche and/or resources. Intestinal tract bacteria such as *Firmicutes* produce metabolites such as butyrate that can inhibit the growth of proteobacteria [9]. Secondly, probiotics and microflora preparations may drive immune development and immunity in the gut helping limit pathogen colonisation. In *Salmonella enterica* infection of the chicken gut, in which it occupies the same niche of the caeca as *Campylobacte*r, a range of probiotics and more complex but undefined microbiota preparations such as Aviguard ™ or Broilact™ have shown good efficacy in reducing pathogen load [25]. Increasingly adopted in Europe, these are cultured products that are unlikely to contain the full complement of species or genera found in the healthy microbiome and are likely to show some bias based upon culture [19]. However, their efficacy against *Campylobacter* infection is more limited as *C. jejuni* has a very low infectious dose {Cawthraw, 1996 #51}and spreads rapidly through flocks, and biosecurity-based interventions are less successful than for *Salmonella* [33].

Furthermore, commercial broiler chickens have no maternal contact, acquiring their early microbiome from the environment of the hatchery and its workers. The early microbiome primarily consists of the bacterial families Enterococcus and Enterobacteriaceae, both of which are opportunistic pathogens of broiler chicks [31]. Poor gut health and dysbiosis are a common problem in broiler production and may be related to a lack of early microbiome diversity [8]. As such, the acquisition of a more diverse chicken microbiome at a younger age could both reduce such infections and lead to enhanced mucosal immunological development. It is evident there is a complex relationship between the chicken microbiota, *C. jejuni* infection and poultry management practice [5, 12, 13, 22].

The idea of microbiome transplants, most notably that of faecal transplants, to improve health and wellbeing has gained considerable traction in both medical and veterinary fields in recent years, though older practices such as transfaunication and microflora products have been used in animal production for many years. The use of transplantation of ‘healthy’ microbiota into broiler chicks offers a possibility to improve gut health and reduce pathogen colonisation, but also to act as an experimental tool to identify phenotypic changes driven by the microbiota and to identify taxa that may be beneficial. Recent production-based studies have shown an association between *C. jejuni* infection status and the microbiome composition [18, 22], though multiple confounding factors in chicken production make identification of direct effects on phenotypes more challenging than in controlled experimental conditions. It is also clear that transplants of the caecal microbiome in chicks can lead to a stable modulation of the microbiome that can reduce pathogen challenge and have clear effects on immune development and function [28].

In this study we used a caecal microbiome transplant (CMT) approach delivered at hatch and determined its efficacy against both direct challenge and a transmission-based challenge model of *C. jejuni* at 21 days of age [4]. We compared efficacy of CMT against that of a commercial microflora preparation (Aviguard) and the effect of treatment on the composition of the caecal microbiome seven days following transfer.

## Methods

### Campylobacter strain and preparation of infection inoculum

*Campylobacter jejuni* M1 was used in all experiments in this study and was kindly provided by Lisa Williams (Hartpury University) [11]. *C. jejuni*, from frozen stock, was plated on Columbia Blood Agar (CBA) and incubated under microaerobic conditions (80% N_2_, 12% CO_2_, 5% O_2_, and 3% H_2_) at 41.5 °C for 48 h. A broth culture was prepared in Mueller Hinton Broth (MHB), followed by incubation overnight under microaerobic conditions, then adjusted to a final level of 10^7^ cfu/ml for infection. Serial 10-fold dilutions of the final MHB culture were made in 1 x Maximum Recovery Diluent (MRD) to 10^− 8^ for viable cell enumeration by a modified Miles & Misra method [23]. All media was provided by LabM, UK.

### Caecal microbiota transplant (CMT) preparation

For preparation of the CMT, approximately 5 ml of digesta were collected aseptically from the caeca each of five 7-week old experimental control animals to create a pooled sample diluted in 200 ml of sterile PBS and coarse filtered through grade 4, 20–25 µM filter to remove large particulates (Whatman, Little Chalfont, UK), These animals were confirmed as being *C. jejuni* negative through cloacal swabbing and bacteriology of caecal and ileal content (described later). All birds were confirmed as being visibly healthy at *post mortem* examination. Caecal content was aliquoted into 5 ml sterile Bijoux containers, Samples snap frozen using liquid nitrogen to prevent deterioration or prolonged aerobic exposure and stored at -80 °C until further use. Prior to use, aliquots were thawed at 37 °C and delivered to recipients within 2 h of thawing.

### Aviguard inoculum preparation

Aviguard (Lallemand, Malvern, UK) was prepared as directed by manufacturer’s instructions for drinking water application. The entire contents of one Aviguard packet (stated treatment sufficiency of 2000 birds) was dissolved in 1 L of sterile ultra-pure water. The prepared Aviguard suspension was inverted for 5 min to ensure compete dispersal. and administered to recipient chicks within one hour of preparation.

### Experimental animals

All work was conducted in accordance with United Kingdom (UK) legislation governing experimental animals under project license PPL P999B8C93 and was approved by the University of Liverpool Animal Welfare and Ethical Review Body prior to the award of this license. All animals held at this site were checked a minimum of twice daily to ensure individual animal health and welfare. Description of experimental housing conditions was as previously described [16, 31].

### Chicks from eggs hatched in unit

For chicks requiring to be hatched within our experimental unit, embryonated Ross 308 broiler eggs were obtained from a commercial hatchery and transported directly to the unit. Eggs were inspected for shell quality and those with catastrophic damage were discarded. Eggs were immersed for 2–3 min in a 1% solution of Ambicide™ (Antec International, Sudbury, UK) maintained at 38–41 °C. Air-dried eggs were wiped with 1% peracetic acid (VWR, Lutterworth, UK) before transfer to a sterile incubator. Eggs were incubated for 21 days at 37.7 °C in an automatic roller incubator (Brinsea, Weston-super-Mare, UK) and candled 7 days after setting to ascertain viability with non-viable eggs discarded. Relative humidity was maintained at 45–55% until day 18 of incubation, then increased to 60–70% and rolling stopped.

Chicks were divided into three treatment groups: CMT treated (0.1–0.2 mL CMT inoculum; *n* = 13), Aviguard treated (0.1–0.2 mL Aviguard; *n* = 13); and controls (0.1–0.2 mL PBS; *n* = 14). Chicks were dosed with treatments by oral gavage within four hours of hatch using a 1 mL sterile syringe. A fourth group was housed separately as aged-matched, unchallenged controls.

## Seeder bird challenge model

Challenge was based on the indirect seeder bird method previously.

### Experimental infection by *C.jejuni* M1

Prior to challenge, all birds were confirmed to be *Campylobacter* free via cloacal swabbing, described below. At 21 days post hatch, two randomly selected birds from each group, excluding the unchallenged control group were dosed by oral gavage with 0.2 mL 10^6^ CFU/mL *C. jejuni* M1 in MHB.

### Assessment of shedding of *C.jejuni*

Cloacal swabs were collected from all birds at 19 (Pre-infection), 23 (2 d.p.i), 26 (5 d.p.i), 29 (8 d.p.i), 31 (10 d.p.i), 33 (12 d.p.i) and 35 (14d.p.i) days post hatch to assess shedding of *C. jejuni*. Briefly, a sterile, cotton swab was inserted 2 cm into the vent and rolled against the mucosal wall. Swabs were streaked onto selective blood-free agar (mCCDA) supplemented with *Campylobacter* enrichment supplement (SV59, Mast Diagnostics, UK) before incubating. The cotton tip of each swab was placed in 2 mL of Exeter enrichment broth containing 5% defibrinated horse blood (Southern Group Laboratories, UK), SV59 and *Campylobacter* growth supplement (FBP; SV61, Mast Diagnostics, UK) and incubated. Following enrichment, samples were vortexed and streaked onto mCCDA. All plates were incubated at 41.5 °C for 48 h in microaerobic conditions before being examined for *C. jejuni* growth. Cloacal swabs were processed within 2 h of collection.

### Necropsy of birds

At 35 days post hatch (14 d.p.i), all birds were culled by cervical dislocation and whole carcass weight recorded. Blood samples were collected via cardiac puncture immediately post-cull. Samples of splenic & liver tissues and caecal & ileal content were aseptically collected.

### Determination of bacterial load

Nine volumes of MRD were added to caecal and ileal content. Enumeration of *C. jejuni* was performed using a modified Miles and Misra method [24] with three replicates of 20 µl at each dilution, onto mCCDA with SV59 supplement. Following microaerobic incubation at 41.5 °C for 48 h, *Campylobacter* colonies were enumerated to calculate CFU/g of caecal contents. In addition, 200 µl were added to 1 ml of Exeter broth and enriched for 48 h.

### Determination of extra-intestinal spread

Liver and spleen samples were weighed and 4 volumes of MRD added before being homogenised in a Stomacher^®^ 80 Biomaster (Seward, UK). Direct detection was assessed by spreading 100 µl of the homogenate onto mCCDA supplemented with SV59 and incubated under microaerobic conditions for 48 h at 41.5 °C. In addition, 200 µl were added to 1 ml of Exeter broth and enriched for 48 h. If direct plating yielded no *Campylobacter* colonies, the enriched samples were plated out and incubated as above.

### Statistical analysis

Data was analyzed using GraphPad Prism 7 for Mac OS X (GraphPad Software Inc., San Diego, USA). All data sets were assessed for normality of distribution using D’Agostino & Pearson normality testing. Pairwise treatment group comparisons of normally distributed data sets were conducted using an Unpaired t test and described using data mean and standard deviation. Pairwise treatment group comparisons of non-normally distributed data sets were conducted using a Mann-Whitney test and described using data median and IQR. For assessment of significance between more than two data sets, Kruskal-Wallis ANOVA testing was performed. P values < 0.05 were considered statistically significant.

### Repeat experiments

#### Experiment 2

Challenge was repeated using birds given CMT (*n* = 19) or controls (*n* = 19) with shedding determined at 2,5, 8 and 12 days post challenge with *post mortem* analysis at 14 days post challenge.

#### Experiment 3

Repeated with smaller groups of CMT (*n* = 10) and untreated controls (*n* = 12). Shedding was determined at 3,5, 7- and 10-days post challenge with *post mortem* analysis at 10 days post challenge.

### Effect of CMT on direct challenge

CMT transplanted (*n* = 20) and control chicks (*n* = 24) were treated and housed as described above. At 21 days each bird was challenged with 10^6^ CFU *C. jejuni* M1 by oral gavage. At 4- and 10-days post challenge 10 CMT and 12 control birds were killed and *C. jejuni* load determined. Additionally at 7- and 10-days post challenge, birds were swabbed to determine faecal shedding.

## Results

### CMT reproducibly reduces *C.jejuni* transmission in broiler chickens

**Fig. 1:**
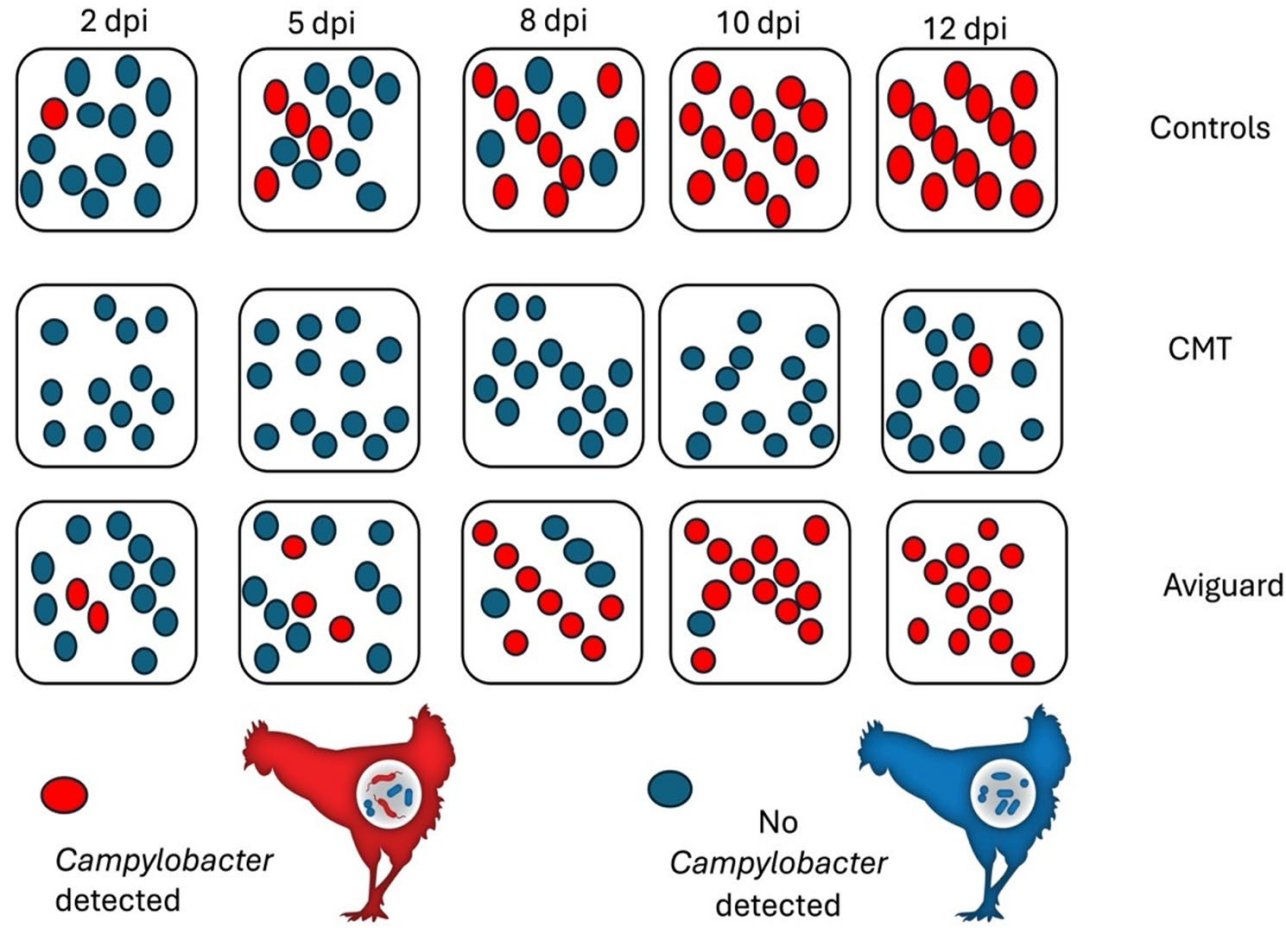
Transmission of *C.* *jejuni* M1 in seeder bird experiment in broilers treated with caecal microbiome transplant (CMT) or Aviguard following challenge of two birds per group at 21 days of age. *C.* *jejuni* colonisation was determined through cloacal swabs enriched in Exeter Broth and plated onto mCCDA agar

**Fig. 2:**
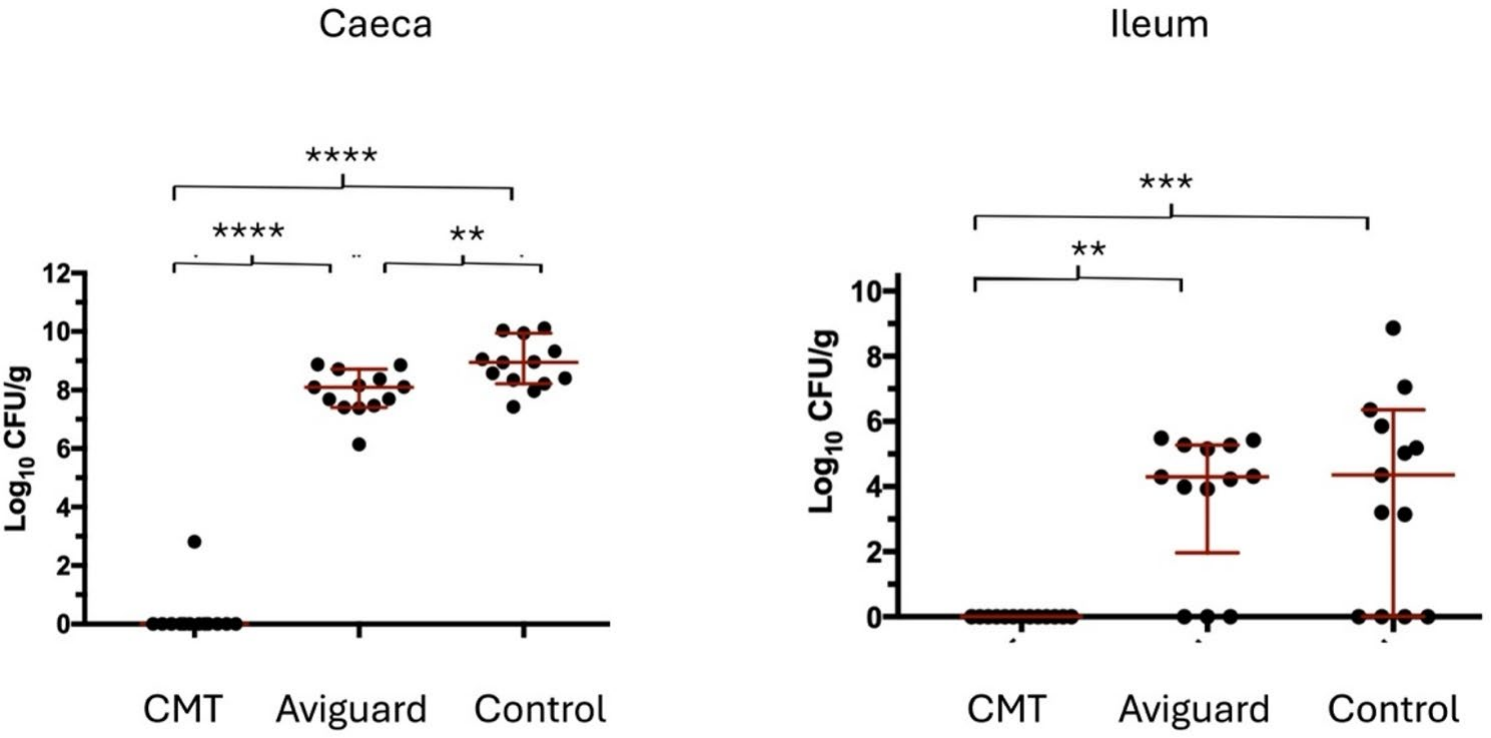
Bacterial load of *C.* *jejuni* M1 in caeca and ileum of individual birds in seeder bird experiment at 14 days post challenge (35 days of age) in birds treat with caecal microbiome transplant (CMT), Aviguard and untreated controls. Quantification on mCCDA agar following dilution in MRD. (*= *P* < 0.05, **=*P* < 0.01, ***_= *P* < 0.005, **** = *P* < 0.001)

**Fig. 3:**
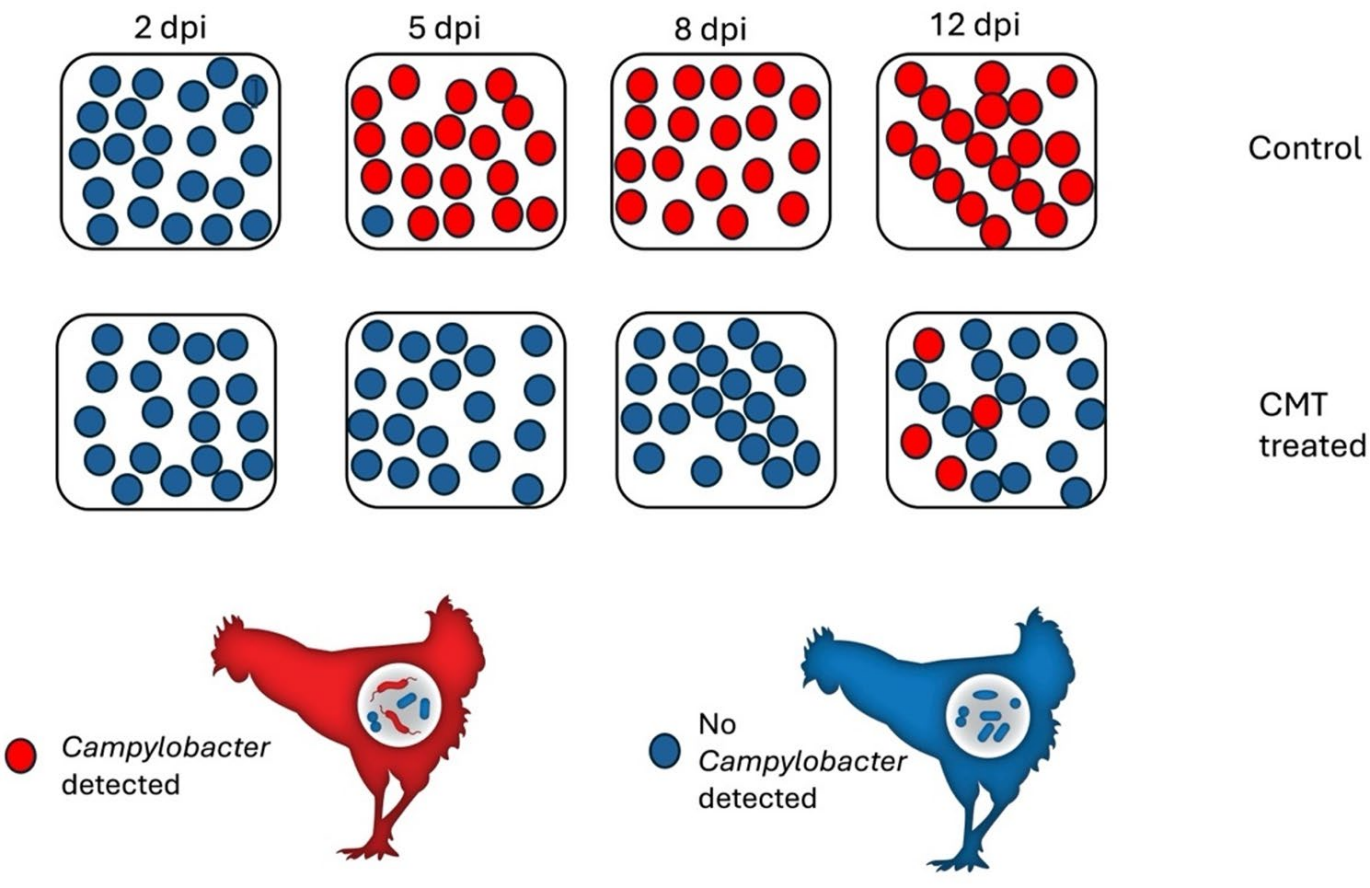
Transmission of *C.* *jejuni* M1 in seeder bird repeat experiment (Experiment 2) in broilers treated with caecal microbiome transplant (CMT) or controls following challenge of two birds per group at 21 days of age. *C.* *jejuni* colonisation was determined through cloacal swabs enriched in Exeter Broth and plated onto mCCDA agar

**Fig. 4:**
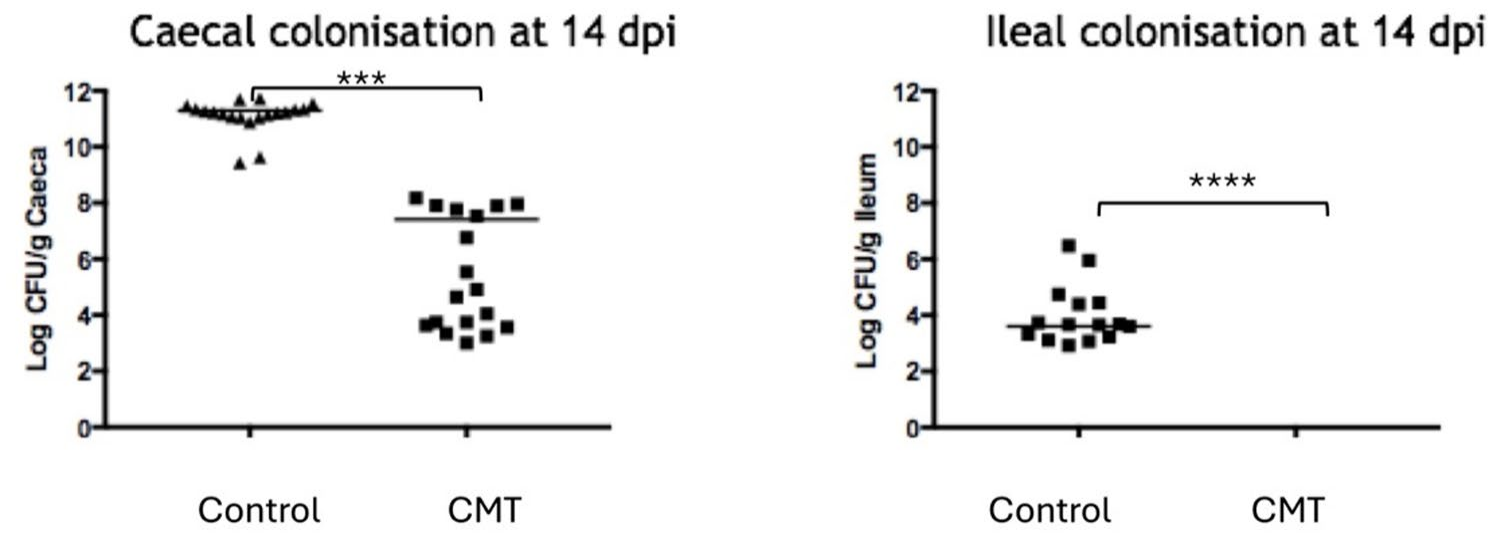
Bacterial load of *C.* *jejuni* M1 in caeca and ileum of individual birds in seeder bird experiment (Experiment 2) at 14 days post challenge (35 days of age) in birds treated with caecal microbiome transplant (CMT) and untreated controls. Quantification on mCCDA agar following dilution in MRD. (*= *P* < 0.05, **=*P* < 0.01, ***_= *P* < 0.005, **** = *P* < 0.001)

CMT reduced the number of birds detected to be faecally shedding *C. jejuni* in the seeder bird challenge model (Fig. 1) and led to a significant reduction in *C. jejuni* colonisation in the caeca and ileum at 15 days post infection (36 days old). Inhibition of colonisation was seen in all three seeder bird challenge experiments, though the effect varied from complete inhibition to a significant reduction in intestinal load of 3 log_10_. (Figures 1, 2, 3 and 4). CMT was significantly more effective than Aviguard both in terms of reduction of transmission (Fig. 1) and in final caecal and ileal load of *C. jejuni* (Fig. 2).

Subsequent repeat experiments reproduced reduction in transmission (Fig. 3) and colonisation (Figs. 4). In experiment 3 no transmission or colonisation was detected in birds that had been given CMT though controls showed levels of colonisation as expected.

### CMT reduces extra-intestinal spread of *C.jejuni*

Tissue samples from both the spleen and liver were collected from all birds at post-mortem examination at 14 days post infection. As with previous samples, all birds in the non-infected control group were negative for *C. jejuni* colonisation and will not be discussed further in any detail.

No birds receiving CMT treatment were positive for *C. jejuni* in either spleen or liver tissue samples collected. In contrast Aviguard-treated and hatchery control groups showed highest frequency of splenic tissue *C. jejuni* infiltration with presence in 5/13 (38.5%) of each population. In the challenge control population, 3/13 (21.1%) were positive for *C. jejuni* in the splenic tissue. Recovery of C. jejuni from livers was more frequent than from spleens in Aviguard-treated birds, with 6/13 (46.2%) positive for *C. jejuni* in liver tissue. The control group had infection than seen for splenic infection, with liver infection in 1/13 (7.7%).

### CMT inhibits rather than prevents infection with *C.jejuni*

**Fig. 5:**
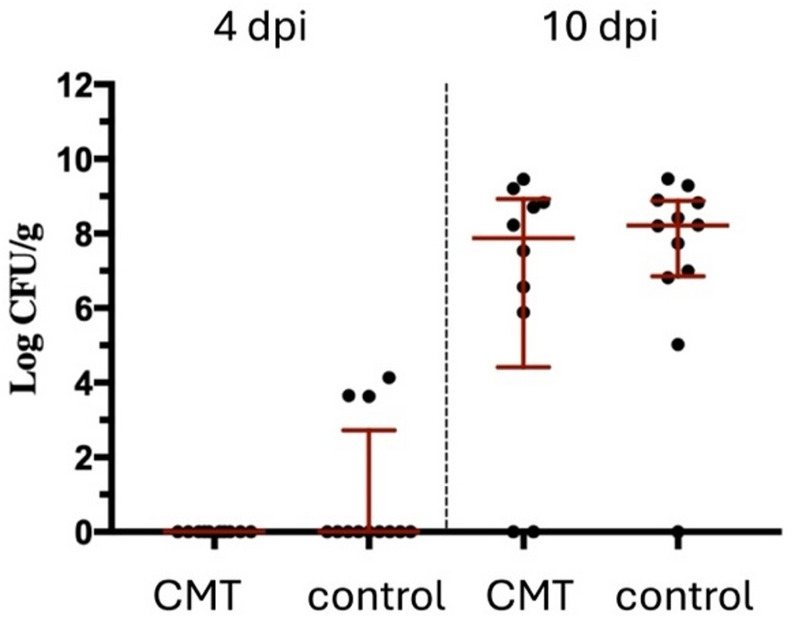
Levels of *C. jejuni* M1 in the caeca of broilers challenged orally with 10^6^ CFU at 7 days of age after receiving caecal microbiome transplant (CMT) or untreated controls

Direct challenge resulted in colonisation of birds receiving CMT, though the establishment of colonisation appears to be inhibited with fewer birds colonized and with fewer *C. jejuni* colonising at 4 days post infection though by 10 days post challenge numbers were similar in the caeca to untreated controls (Fig. 5). Fewer CMT birds (3/10) were shedding *C. jejuni* than controls (7/12) at 7 days post infection. Together these data suggest that although CMT does not prevent colonisation with *C. jejuni* following direct challenge there is a degree of inhibition that manifests as reductions in early bacterial numbers and faecal shedding.

## Discussion

Here we have shown that early transplantation of a mature caecal microbiome in broiler chicks significantly reduces transmission and colonisation of the foodborne pathogen *C. jejuni.* Treated birds had colonisation levels in their caeca significantly reduced at slaughter age, thereby reducing the risk of *C. jejuni* entering the food chain. The reduction in bacterial numbers is reproducible and greater than that achieved in production age birds by experimental vaccines and other interventions, with the levels reduced to those predicted by modelling and risk analysis to significantly reduce human infection [14]. Transplantation leads to a change in composition of the intestinal microbiome, both in terms of the numbers and diversity in bacterial taxa. These data show that transmission and colonisation of *Campylobacter* can be significantly reduced by modulation of the intestinal microbiome opening up a range of possibilities to its control through microbial interventions and by prebiotic feed additives. Whilst in a direct challenge model CMT ultimately leads to a limited reduction in caecal load, it still acts to delay colonisation and faecal shedding. Another recent study using similar methods found inhibition in direct challenge, but not in a seeder bird infection [26]. As the authors describe this may be a consequence of differences in the transplant material sourced from commercial birds and in the chickens themselves. Furthermore, a different challenge strain of *C. jejuni* may behave differently as we have previously shown a degree of heterogeneity in the infection biology of *C. jejuni* strain [17].

Developing controls for *Campylobacter* in the chicken require a better understanding of infection within the gut ecosystem including that of the pathogen, host response and microbiome. The advent of pathogen genomic-based approaches has allowed us to determine the major bacterial factors involved in colonisation [6], but our understanding of the host response, the bacterial metabolic pathways during *C. jejuni* infection and that of the role of the microbiome has considerable gaps. *C. jejuni* was previously considered a commensal in the chicken. However, in recent years it has become apparent that its relationship is somewhere between that of part of the microbiome and a pathogen. It is clear that *C. jejuni* initially causes damage to the intestinal barrier and elicits an inflammatory immune response [1, 2, 16], and that some strains are capable of spread beyond the intestinal tract to the spleen, liver and in some cases to muscle tissue. Although the host mounts an immune response, it does not clear infection within the ceca, a response considered ‘tolerogenic’ [15]. The nature of the response with specific antibody and Th17 responses in the intestinal tract appears to be one that primarily restricts *Campylobacter* to the gut without preventing colonisation or leading to clearance [10, 21, 30], whereas other intestinal infections such as *Salmonella* and *Eimeria* lead to clearance largely involving Th1-mediated responses [36]. It is also clear that the microbiome influences infection and indeed that *Campylobacter* infection leads to changes within the microbiome. Large scale studies have shown an association between specific bacterial taxa and infection status and that changes in the developing intestinal microbiome may be related to increased susceptibility or ‘a window of opportunity’ for *C. jejuni* infection along with the decline in maternal antibody [5, 18]. Infection with *C. jejuni* also causes a perturbance in the microbiome structure [32, 35]. Taken together there is undoubtedly a complex interaction of host response, pathogen biology and microbiome that act to either facilitate or restrict *C. jejuni* colonisation, but there are also fundamental gaps in our understanding that make the development of rational interventions challenging. Current scientific opinion suggests that vaccination or feed-based interventions are the most likely to lead to reducing the *Campylobacter* burden in chicken production. Recently estimates of the levels of *C. jejuni* reduction in broiler production needed to reduce human infection have been revised to reflect a considerably lower effect than previous estimates with a 3-log_10_ reduction in broiler caecal levels needed to lower human infections by 58%. Such reductions are a challenge to producing an effective vaccine to an enteric pathogen in what are immunologically naïve neonatal animals slaughtered at around six weeks of age. Current experimental vaccines tending to only show reproducible efficacy at an older age and rarely at 3‐log_10_ reduction levels. On this basis rational and targeted microbial-based interventions may offer the best approach.

There have been many attempts to use conventional probiotic and competitive exclusion-based approaches towards *Campylobacte*r control, though their success is mixed compared to *Salmonella*, where exclusion using single probiotic strains or more complex preparations such as Aviguard have been proven to be successful in reducing the burden of infection. Whilst many studies have shown in vitro efficacy for a range of probiotic candidates, notably *Lactobacillus* where there is good evidence of immunomodulation [34], their translation into in vivo efficacy is less apparent [3, 7]. In this study we have used a single early intervention with a whole caecal microbiome consisting of close to 500 individual amplified sequence variants which are considered a proxy for individual taxa [27]. We have been able to demonstrate many of these taxa have colonised the chicken gut leading to modification of the caecal and ileal microbiomes. In turn this reduces infection in a seeder bird model. This may be regarded as a more natural model of *Campylobacter* challenge closer to that seen in commercial broiler flocks where infection is spread by faecal-oral transmission via the coprophagic nature of chickens. In this model the infectious dose will be lower, but variable, and transmission will rely on a range of factors including frequency of bird-to-bird contact and amount of *C. jejuni* shed in faeces and the amount of faecal material ingested by other birds. Indeed, the stochastic nature of the model means variability is likely to occur which may go some way to explaining the variation in reduction of transmission from complete to reduced levels of transmission and colonisation. At the detail we have examined there is no ‘smoking gun’ of individual taxa that appear to drive protection to enteric infection, though notably CMT and to a lesser extent Aviguard does reduce the proportion of Enterobacteriaceae in the microbiome, high levels of which have been implicated in susceptibility to *Campylobacter* colonisation and associated with poor gut health [32]. As Aviguard is a cultured product it will be both less diverse than CMT and it may be that adaptation to the in vitro environment has reduced the ability of components to colonise in vivo. The ability to colonise the chicken gut is a key to any probiotic or microbial intervention and the use of CMT coupled with sequencing-based microbiome analysis can identify taxa found both in the ‘healthy’ gut microbiome and transplanted to give a beneficial phenotype [27], thereby offering a rational approach to identify candidates.

Perhaps the biggest question is why transmission is reduced, something we do not address in this study. We may consider this a direct consequence of the transplanted microbiota excluding *Campylobacter* through occupation of the same niche, competition for nutrients or through the production of metabolites. However, the evidence for successful CE against *Campylobacter* is mixed. This opens the possibility that an early diverse microbiome better drives immune development in the gut. In contrast whilst single probiotics can reduce pathogen colonisation, the effect diminishes following removal from diet [20], whereas in this study effects are apparent some three weeks following administration. We know that exposure to maternal faeces or the provision of an ‘artificial microbiome’ increases immunoglobulin responses and that the microbiome is associated with development of the T cell repertoire [37]. Although our understanding of the avian immune response in campylobacteriosis has improved considerably in recent years, our knowledge of what mechanisms lower *C. jejuni* levels in the gut is rudimentary. We know antibody responses are associated with clearance from the small intestine, but that antibody has only a limited effect on caecal colonisation. There also appears to be a clear role for regulatory and Th17 cells in maintenance of gut integrity and homeostasis during *C. jejuni* infection, yet these cell types and their location and development are poorly understood in avian species. It is tempting to speculate an immunological role here, but much more evidence is needed to make such a link, though the prevention of extraintestinal infection following CMT lends weight to a stronger intestinal barrier, though of course fewer birds are colonised. It is clear, as in mammals, that the microbiome is a key driver of immunological development. The acquisition of a diverse early microbiome is almost certainly a major factor in mucosal immune development of the chicken. The use of microbiome transplants or indeed artificial microbiomes represents a useful tool to determine how and better characterise the function of T cell populations in infection and gut health as well as a platform to develop rational microbial-based interventions against *C. jejuni* in chicken production.

In conclusion, early modification of the broiler chick microbiome via transplantation leads to a more diverse microbiome, less prevalent in Enterococcus and Enterobactericiae, but crucially leads to a phenotype less amenable to colonisation with and transmission of *C. jejuni* with highly significant lower levels of bacteria in the caeca at slaughter age. This shows unequivocally the potential for microbial-based interventions in reducing the burden of campylobacteriosis. The use of transplants, though not a practical solution for commercial production, is a valuable tool in rational approaches to identify protective bacterial species to act as probiotics or in defined consortia, also aid understanding the role of the microbiome in immune development and function without the difficulty and extreme costs of germ-free chicken studies.

## Conclusions

We have shown that a microbiome transplant can reduce both transmission and final caecal levels of *C. jejuni* in broiler chickens. Whilst a caecal transplant may not be practical for translation to industry, it shows proof-of-principle that microbial interventions can be effective in controlling *C. jejuni* in broiler chickens. Furthermore, it offers a route for rational discovery of effective probiotics based on function and a low-cost platform to understand how the microbiota drives immune development.

## Data Availability

Raw data will be made available on reasonable request.

## References

[1] Awad WA, Molnar A, Aschenbach JR, Ghareeb K, Khayal B, Hess C, Liebhart D, Dublecz K, Hess M. Campylobacter infection in chickens modulates the intestinal epithelial barrier function. Innate Immun. 2015;21:151–60. 24553586 10.1177/1753425914521648

[2] Awad WA, Ruhnau D, Hess C, Hess M. Campylobacter jejuni increases the paracellular permeability of broiler chickens in a dose-dependent manner. Poult Sci. 2020;99:5407–14. 33142457 10.1016/j.psj.2020.08.014 PMC7647851

[3] Balta I, Butucel E, Stef L, Pet I, Gradisteanu-Pircalabioru G, Chifiriuc C, Gundogdu O, McCleery D, Corcionivoschi N. Anti-Campylobacter Probiotics: Latest Mechanistic Insights. Foodborne Pathog Dis. 2022;19:693–703. 35905047 10.1089/fpd.2022.0039 PMC9595622

[4] Chaloner G, Wigley P, Humphrey S, Kemmett K, Lacharme-Lora L, Humphrey T, Williams N. Dynamics of dual infection with Campylobacter jejuni strains in chickens reveals distinct strain-to-strain variation in infection ecology. Appl Environ Microbiol. 2014;80:6366–72. 25107966 10.1128/AEM.01901-14 PMC4178652

[5] Connerton PL, Richards PJ, Lafontaine GM, O’Kane PM, Ghaffar N, Cummings NJ, Smith DL, Fish NM, Connerton IF. The effect of the timing of exposure to Campylobacter jejuni on the gut microbiome and inflammatory responses of broiler chickens, *Microbiome*, 2018;6:88. 10.1186/s40168-018-0477-5 PMC5948730 29753324

[6] de Vries SP, Gupta S, Baig A, Wright E, Wedley A, Jensen AN, Lora LL, Humphrey S, Skovgard H, Macleod K, Pont E, Wolanska DP, L’Heureux J, Mobegi FM, Smith DGE, Everest P, Zomer A, Williams N, Wigley P, Humphrey T, Maskell DJ, Grant AJ. Genome-wide fitness analyses of the foodborne pathogen Campylobacter jejuni in in vitro and in vivo models. Sci Rep. 2017;7:1251. 28455506 10.1038/s41598-017-01133-4 PMC5430854

[7] Deng W, Dittoe DK, Pavilidis HO, Chaney WE, Yang Y, Ricke SC. Current Perspectives and Potential of Probiotics to Limit Foodborne Campylobacter in Poultry. Front Microbiol. 2020;11:583429. 33414767 10.3389/fmicb.2020.583429 PMC7782433

[8] Diaz Carrasco JM, Casanova NA, Fernandez Miyakawa ME. Microbiota, gut health and chicken productivity: What is the connection?, *Microorganisms*, 2019;7. 10.3390/microorganisms7100374 PMC6843312 31547108

[9] Eeckhaut V, Van Immerseel F, Croubels S, De Baere S, Haesebrouck F, Ducatelle R, Louis P, Vandamme P. Butyrate production in phylogenetically diverse Firmicutes isolated from the chicken caecum. Microb Biotechnol. 2011;4:503–12. 21375722 10.1111/j.1751-7915.2010.00244.x PMC3815262

[10] Flaujac Lafontaine GM, Richards PJ, Connerton PL, O’Kane PM, Ghaffar NM, Cummings NJ, Fish NM, Connerton IF. Prebiotic Driven Increases in IL-17A Do Not Prevent Campylobacter jejuni Colonization of Chickens. Front Microbiol. 2019;10:3030. 32010094 10.3389/fmicb.2019.03030 PMC6972505

[11] Friis C, Wassenaar TM, Javed MA, Snipen L, Lagesen K, Hallin PF, Newell DG, Toszeghy M, Ridley A, Manning G. and D. W. Ussery. Genomic characterization of Campylobacter jejuni Strain M1. PLoS ONE. 2010;5(8):e12253. 10.1371/journal.pone.0012253 PMC2928727 20865039

[12] Han Z, Willer T, Li L, Pielsticker C, Rychlik I, Velge P, Kaspers B, Rautenschlein S. Influence of the gut microbiota composition on Campylobacter jejuni colonization in chickens. Infect Immun. 2017;85(11). 10.1128/iai.00380-17. 10.1128/IAI.00380-17 PMC5649013 28808158

[13] Han Z, Willer T, Pielsticker C, Gerzova L, Rychlik I, Rautenschlein S. Differences in host breed and diet influence colonization by Campylobacter jejuni and induction of local immune responses in chicken. Gut Pathog. 2016;8:56. 27843492 10.1186/s13099-016-0133-1 PMC5105272

[14] Hazards EP on, Biological K, Koutsoumanis A, Allende A, Alvarez-Ordonez D, Bolton S, Bover-Cid R, Davies A, De Cesare L, Herman F, Hilbert R, Lindqvist M, Nauta L, Peixe G, Ru M, Simmons P, Skandamis E, Suffredini T, Alter M, Crotta J, Ellis-Iversen M, Hempen W, Messens and M. Chemaly. Update and review of control options for Campylobacter in broilers at primary production, EFSA J. 2020;18:e06090. 10.2903/j.efsa.2020.6090 PMC7448041 32874298

[15] Hermans D, Pasmans F, Heyndrickx M, Van Immerseel F, Martel A, Van Deun K, Haesebrouck F. A tolerogenic mucosal immune response leads to persistent Campylobacter jejuni colonization in the chicken gut. Crit Rev Microbiol. 2012;38:17–29. 21995731 10.3109/1040841X.2011.615298

[16] Humphrey S, Chaloner G, Kemmett K, Davidson N, Williams N, Kipar A, Humphrey T, Wigley P. Campylobacter jejuni is not merely a commensal in commercial broiler chickens and affects bird welfare, *mBio*, 2014;5:e01364-14. 10.1128/mBio.01364-14 PMC4161246 24987092

[17] Humphrey S, Lacharme-Lora L, Chaloner G, Gibbs K, Humphrey T, Williams N, Wigley P. Heterogeneity in the Infection Biology of Campylobacter jejuni Isolates in Three Infection Models Reveals an Invasive and Virulent Phenotype in a ST21 Isolate from Poultry. PLoS ONE. 2015;10:e0141182. 26496441 10.1371/journal.pone.0141182 PMC4619714

[18] Ijaz UZ, Sivaloganathan L, McKenna A, Richmond A, Kelly C, Linton M, Stratakos AC, Lavery U, Elmi A, Wren BW, Dorrell N, Corcionivoschi N, Gundogdu O. Comprehensive Longitudinal Microbiome Analysis of the Chicken Cecum Reveals a Shift From Competitive to Environmental Drivers and a Window of Opportunity for Campylobacter. Front Microbiol. 2018;9:2452. 30374341 10.3389/fmicb.2018.02452 PMC6196313

[19] Kerr AK, Farrar AM, Waddell LA, Wilkins W, Wilhelm BJ, Bucher O, Wills RW, Bailey RH, Varga C, McEwen SA, Rajic A. A systematic review-meta-analysis and meta-regression on the effect of selected competitive exclusion products on Salmonella spp. prevalence and concentration in broiler chickens. Prev Vet Med. 2013;111:112–25. 23731553 10.1016/j.prevetmed.2013.04.005

[20] Khan S, Chousalkar KK. Salmonella Typhimurium infection disrupts but continuous feeding of Bacillus based probiotic restores gut microbiota in infected hens. J Anim Sci Biotechnol. 2020;11:29. 32211190 10.1186/s40104-020-0433-7 PMC7087389

[21] Lacharme-Lora L, Chaloner G, Gilroy R, Humphrey S, Gibbs K, Jopson S, Wright E, Reid W, Ketley J, Humphrey T, Williams N, Rushton S, Wigley P. B lymphocytes play a limited role in clearance of Campylobacter jejuni from the chicken intestinal tract. Sci Rep. 2017;7:45090. 28332622 10.1038/srep45090 PMC5362810

[22] McKenna A, Ijaz UZ, Kelly C, Linton M, Sloan WT, Green BD, Lavery U, Dorrell N, Wren BW, Richmond A, Corcionivoschi N, Gundogdu O. Impact of industrial production system parameters on chicken microbiomes: mechanisms to improve performance and reduce Campylobacter. Microbiome. 2020;8:128. 32907634 10.1186/s40168-020-00908-8 PMC7488076

[23] Miles AA, Misra SS, Irwin JO. The estimation of the bactericidal power of the blood. J Hyg (Lond). 1938a;38:732–49. 20475467 10.1017/s002217240001158x PMC2199673

[24] Miles AA, Misra SS, Irwin JO. The estimation of the bactericidal power of the blood. J Hygiene. 1938b;38:732–49. 20475467 10.1017/s002217240001158x PMC2199673

[25] Nakamura A, Ota Y, Mizukami A, Ito T, Ngwai YB, Adachi Y. Evaluation of aviguard, a commercial competitive exclusion product for efficacy and after-effect on the antibody response of chicks to Salmonella. Poult Sci. 2002;81:1653–60. 12455592 10.1093/ps/81.11.1653

[26] Pang J, Beyi AF, Looft T, Zhang Q, Sahin O. Fecal Microbiota transplantation reduces Campylobacter jejuni colonization in young broiler chickens challenged by oral gavage but not by seeder birds. Antibiot (Basel). 2023;12(10):1503. 10.3390/antibiotics12101503 PMC10604036 37887204

[27] Pottenger S, Watts A, Wedley A, Jopson S, Darby AC, Wigley P. Timing and delivery route effects of cecal microbiome transplants on Salmonella Typhimurium infections in chickens: potential for in-hatchery delivery of microbial interventions. Anim Microbiome. 2023;5:11. 36788638 10.1186/s42523-023-00232-0 PMC9926694

[28] Ramirez GA, Richardson E, Clark J, Keshri J, Drechsler Y, Berrang ME, Meinersmann RJ, Cox NA. and B. B. Oakley. Broiler chickens and early life programming: microbiome transplant-induced cecal community dynamics and phenotypic effects. PLoS ONE. 2020;15(11):e0242108. 10.1371/journal.pone.0242108 PMC7665843 33186366

[29] Rantala M, Nurmi E. Prevention of the growth of Salmonella infantis in chicks by the flora of the alimentary tract of chickens. Br Poult Sci. 1973;14:627–30. 4759990 10.1080/00071667308416073

[30] Reid WD, Close AJ, Humphrey S, Chaloner G, Lacharme-Lora L, Rothwell L, Kaiser P, Williams NJ, Humphrey TJ, Wigley P, Rushton SP. Cytokine responses in birds challenged with the human food-borne pathogen Campylobacter jejuni implies a Th17 response. R Soc Open Sci. 2016;3:150541. 27069644 10.1098/rsos.150541 PMC4821255

[31] Richards P, Fothergill J, Bernardeau M, Wigley P. Development of the Caecal Microbiota in Three Broiler Breeds. Front Vet Sci. 2019;6:201. 31294039 10.3389/fvets.2019.00201 PMC6603203

[32] Sakaridis I, Ellis RJ, Cawthraw SA, van Vliet AHM, Stekel DJ, Penell J, Chambers M, La Ragione RM, Cook AJ. Investigating the association between the caecal microbiomes of broilers and campylobacter burden, *Front Microbiol*, 2018;9:927. 10.3389/fmicb.2018.00927 PMC5972209 29872425

[33] Schneitz C, Hakkinen M. The efficacy of a commercial competitive exclusion product on Campylobacter colonization in broiler chickens in a 5-week pilot-scale study. Poult Sci. 2016;95:1125–8. 26944963 10.3382/ps/pew020 PMC4957530

[34] Taha-Abdelaziz K, Astill J, Kulkarni RR, Read LR, Najarian A, Farber JM, Sharif S. In vitro assessment of immunomodulatory and anti-Campylobacter activities of probiotic lactobacilli. Sci Rep. 2019;9:17903. 31784645 10.1038/s41598-019-54494-3 PMC6884649

[35] Thibodeau A, Fravalo P, Yergeau E, Arsenault J, Lahaye L, Letellier A. Chicken Caecal Microbiome Modifications Induced by Campylobacter jejuni Colonization and by a Non-Antibiotic Feed Additive. PLoS ONE. 2015;10:e0131978. 26161743 10.1371/journal.pone.0131978 PMC4498643

[36] Wigley P. Salmonella enterica in the Chicken: How it has Helped Our Understanding of Immunology in a Non-Biomedical Model Species. Front Immunol. 2014;5:482. 25346731 10.3389/fimmu.2014.00482 PMC4193332

[37] Zenner C, Hitch TCA, Riedel T, Wortmann E, Tiede S, Buhl EM, Abt B, Neuhaus K, Velge P, Overmann J, Kaspers B, Clavel T. Early-life immune system maturation in chickens using a synthetic community of cultured gut bacteria, *mSystems*,2021;6. 10.1128/mSystems.01300-20 PMC8269260 34006629

